# Barriers to and motives for engagement in an exercise-based cardiac rehabilitation programme in Ireland: a qualitative study

**DOI:** 10.1186/s12875-022-01637-7

**Published:** 2022-02-11

**Authors:** Alison Bourke, Vikram Niranjan, Raymond O’Connor, Catherine Woods

**Affiliations:** 1 School of Medicine, University of Limerick, Limerick, Ireland; 2 School of Public Health, Physiotherapy and Sport Sciences, University College Dublin, Dublin, Ireland; 3 Health Research Institute, University of Limerick, Limerick, Ireland; 4 Department of Physical Education and Sports Sciences, University of Limerick, Limerick, Ireland

**Keywords:** Primary health care, cardiac rehabilitation, Barriers, Motives, National exercise referral framework

## Abstract

**Background:**

Insufficient physical activity (PA) is a leading risk factor for premature death worldwide. Ireland’s public healthcare system, the Health Service Executive (HSE), has supported the development of the National Exercise Referral Framework (NERF) to tackle low levels of PA amongst those with non-communicable diseases (NCDs). ‘NERF centres’ are medically supervised PA programmes across Ireland that have established referral pathways with local hospitals and general practitioners. ULMedX is one such NERF centre offering exercise-based cardiac rehabilitation (EBCR) with the aim of intervention development to reduce early drop-out and maximise adherence for optimal health benefits.

**Aim:**

The purpose of this research was to identify the major factors influencing participants’ adherence and early drop-out at ULMedX. Exploring areas for future development were also prioritised.

**Design & setting:**

Qualitative interviews were conducted with long-term attenders and people who have dropped out (PWDO) from ULMedX.

**Methods:**

Guided by the Theory of Planned Behaviour the 1–1 semi-structured interviews were performed, transcribed, and evaluated through thematic analysis.

**Results:**

Analysis was performed on 14 participants (50% female; mean age 67.3 years), comprising long-term attenders (*n* = 7; 13-month duration, 64% of classes) and PWDO (*n* = 7; 2.8-month duration, 22% of classes). Three major factors affecting adherence and drop-out were identified: social support, perceived outcomes from participation and practical barriers to attendance. Areas for future development included the provision of evening and advanced classes, psychological support, more exercise variety, more educational seminars and new members start as their own group.

**Conclusion:**

The findings suggest participants at ULMedX are more likely to have had a better experience and commit to the programme if they believed involvement would benefit their physical and mental health, increase their exercise motivation by engendering a positive attitude to exercise, and that the ability to attend was within their control. Future interventions at ULMedX should have their structures centred around these motives for engagement. ULMedX should also test the participant recommendations to overcome the common barriers to adherence.

## Background

Although regular physical activity (PA) is increasingly being recognised as a critical factor for good health and disease management, the global prevalence of insufficient PA is 27·5% [1], and physical inactivity is still the fourth leading global risk factor for mortality [2]. Cardiovascular disease (CVD) is also one of the most common causes of death and disability worldwide [3] and accounts for 30.1% of all deaths in Ireland [4, 5]. It is also one of the diseases most commonly associated with physical inactivity [6, 7]. Exercise-based cardiac rehabilitation (EBCR) programmes are regarded by many as essential in the management of CVD [8, 9] and usually consists of exercise training alone or in combination with psychosocial or educational interventions [9]. These programmes may be hospital-based or community-based. This study focuses on one EBCR centre within a community setting in Ireland.

The high quality of evidence supporting the use of PA for primary and secondary prevention of several chronic conditions including CVD has led to the setting up of the Irish National Exercise Referral Framework (NERF) [10]. ‘ULMedX’ is a community-based chronic illness rehabilitation centre established at the University of Limerick in 2017, in line with the NERF policy [10]. The centre offers medically supervised exercise classes and educational workshops, as well as peer support through social activities after class. It currently providesrs an EBCR programme for patients with CVD [8, 9].

Despite extensive evidence advocating the integration of EBCR programmes into the treatment plans for CVD, participant uptake (agreeing to be referred to an EBCR programme and then attending the first appointment) can range from 14 to 35% [11, 12]. Furthermore, among those who then participate in the EBCR programmes, adherence levels can fall as low as 37% [13].

Attendance records at ULMedX in 2018 indicated a gradual decline in adherence as the year progressed and an early drop-out. Adherence was 60%, 57% and 37% in January 2018, March 2018, and January 2019, respectively. In addition, 75% of all individuals who dropped out of the programme did so before their twelfth week at ULMedX. Many factors have been shown to influence non-adherence in similar programmes. These include, but are not limited to, low social support, longer travel times, older age, and low income [14].

This research aimed to identify the reasons for people with established and stable cardiovascular disease disengaging with ULMedX despite having very good medical reasons to continue with it. The specific objectives were to identify barriers and motives for adherence, as well as suggestions for future development of this one EBCR programme located in the Mid-West of Ireland.

To the best of our knowledge, this is the first study in Ireland to include the experiences of those who have dropped out of an EBCR programme in their analysis. It is also the first study to include recommendations for future developments in an EBCR programme from both long-term attenders and those who have dropped out.

This information will be used to develop an intervention to increase adherence and reduce early drop-out at ULMedX. The information obtained may also be used by other NERF centres and EBCR programmes currently being set up across Ireland.

## Methods

### Study design

Qualitative interviews were conducted to address the study objectives. This involved 1–1 semi-structured interviews with long-term attenders and people who have dropped out (PWDO) from one EBCR programme in Ireland named ‘ULMedX’. Participants identified their motives and barriers to commitment, expectations, and perception of the ULMedX programme. Interviews were recorded, transcribed, and evaluated using thematic analysis.

### Setting

This study was set in the Mid-West of Ireland. Interviews were conducted from a private room within the University of Limerick Sports arena where ULMedX classes occur (Table 1). Telephone interviews were also performed from within this room.

**Table 1:** ULMedX EBCR

**Description of the programme** ULMedX has a user-pay EBCR programme located within a third level educational institution and a community setting, with over 80 visits per week. The primary mission of the ULMedX EBCR programme is to transform the lives of people with CVD through physical activity-based rehabilitation. The service is delivered by trained personnel from the sports centre along with academic staff, graduate, and undergraduate students. All exercise sessions adhere to the same structure of warm-up, aerobic exercise, resistance exercise and cool-down, with a post-class refreshment, in total 90 min duration. Participant's adherence is monitored by the number of classes attended, which the students manually recorded. Classes are supervised by trained staff at a ratio of 1:15. Participants are encouraged to attend two classes per week. They are also advised to attend the same sessions every week to foster social support and habit formation. Educational workshops on exercise and nutrition are scheduled regularly throughout the year for the participants.	

### Participants and recruitment

ULMedX participants are referred from one of the University of Limerick Hospital Group (ULHG) locations or by general practitioners in the Mid-West of Ireland.

Current ULMedX participants were informed about the study directly by the primary researcher during ULMedX classes. The primary researcher used A poster to facilitate this recruitment. Those interested in participating gave their name and contact information to the primary researcher. After excluding those who did not meet the criteria (Table 2), a purposeful sample of long-term attenders was chosen to reflect the breadth of individuals who consistently engaged with ULMedX. This sample contained a mix of gender, age, and fitness levels.

**Table 2:** Inclusion and exclusion criteria

	Long-term attenders (LT)	People who have dropped out (PWDO)
**Inclusion**	Adults with an established and stable cardiovascular disease. Attending the EBCR programme regularly for at least six months. This is the accepted time frame for regulating behaviour change [15].	Adults with an established and stable cardiovascular disease. Previously attended ULMedX but have not attended a programme session in the previous three months. Drop-out was defined as the withdrawal from class and described elsewhere [16].
**Exclusion**	Individuals with symptomatic or uncontrolled cardiovascular conditions. Absolute contraindications to exercise [17]. Inability to provide informed consent.	

PWDO who met the criteria were identified upon reviewing ULMedX attendance records. Potential participants were informed about the research over the phone. For the PWDO interested in taking part, an in-person or telephone interview was offered. All PWDO who agreed to take part were interviewed and included in this analysis.

All Individuals expressing an interest to participate in the study were provided with a plain language statement. They were also given the opportunity to ask the primary researcher questions and to withdraw from the study at any point. Written informed consent was required from all participants before the interviews took place.

### Data collection

**Fig. 1:**
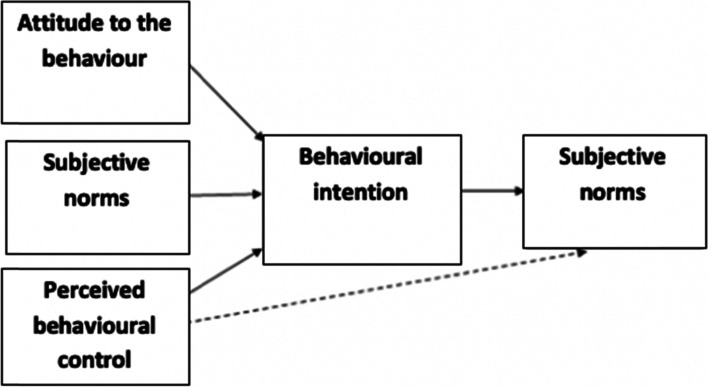
The theory of planned behaviour- proposed determinants of behaviour

Interview questions were guided by existing literature, the experience of the research team and the ‘Theory of Planned Behaviour (TPB)’ (Fig. 1) [18]. One interview script was used for both PWDO and long-term attenders, however an additional set of questions were given to the PWDO to make known their reasons for dropping out. All interviews lasted between 20 and 60 min and were audio-recorded and transcribed manually by one researcher (AB). The reviewer noted of emerging topics and amendments for future interviews when necessary [19]. Data gathering ceased after 14 interviews once data saturation was reached [20].

### Data analysis

**Fig. 2:**
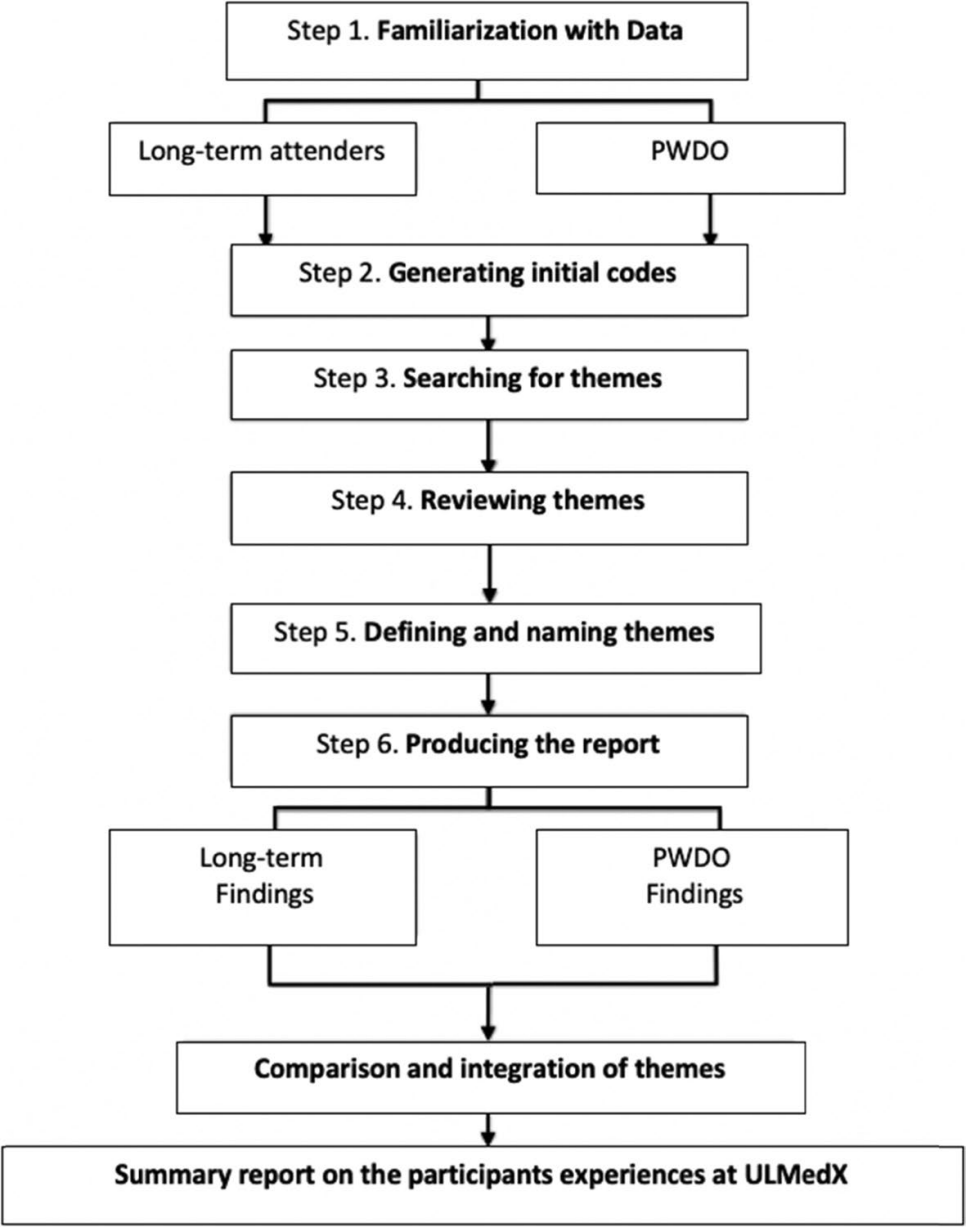
A step by step illustration of thematic analysis. *PWDO* participants who have dropped out

The researchers analysed the data manually using the thematic analysis approach described by Braun and Clark (Fig. 2) [21]. Initial codes and themes were performed manually by the primary research. The findings were reviewed and amended by two additional researchers, one of whom was an external researcher who did not have any previous contact with the EBCR programme.

Four thematic areas were constructed based on study objectives and the theory of planned behaviour. These included themes pertaining to ‘Support at ULMedX, Benefits from ULMedX, Motivation to attend ULMedX and Barriers towards ULMedX’. In addition to this, the participants identified ‘Areas for Future Development’ to improve adherence and reduce early drop-out at ULMedX. The researchers then jointly developed a thematic analysis framework beginning with the four themes which were further classified and clustered under sub-themes and codes, to organise data more strategically. For example, the thematic area ‘Support at ULMedX’ was classified into sub-theme like ‘professional, family, peer’ and further broken down into codes like ‘GP recommendation, family support, peer encouragement’ (Table 4 below). The codes were updated continuously as they emerged during the coding process. Disagreements between researchers were resolved by reviewing the subset of transcripts multiple times and then engaging in discussions to determine what truly represents the data. The framework was subsequently revised to incorporate new and refined codes. The process of refining, applying, and re-refining the thematic analysis framework was repeated until no new codes were generated.

The final thematic analysis framework was applied to each transcript. Each transcript was 

systematically reviewed, highlighting meaningful passages of text, and selecting and attaching an appropriate code from the final analytical framework. These themes were then compared to examine relationships, specifically the multiple relationships and interrelationships that exist between the themes themselves. Data were also presented in the form of descriptive accounts, and these consisted of illustrative quotes taken from the interviews. These are shown in the results chapter.

## Results

### Participants characteristics’

Out of the fifteen participants selected, fourteen were included in this report. One participant (LT attender) did not partake in the interview due to ongoing conflicting hospital appointments during the data collection period (Table 3).

**Table 3:** Participant’s characteristics

	All participants (***n*** = 14)	Long-term attender (***n*** = 7)	PWDO (***n*** = 7)
Variables	Mean (SD)	Mean (SD)	Mean (SD)
**Age** *(years)*	67.3 *(10.4)*	65.7 *(10.2)*	68.9 *(10.3)*
**Gender** *Female (%)*	50	57	43
*Male (%)*	50	43	57
**Work Status** *Payment (%)*	43	43	43
*No payment (%)*	14	0	28.5
*Retired (%)*	43	57	28.5
**Distance from ULMedX** *(km)*	31.6 *(23.5)*	22.01 *(15.3)*	41.1 *(26.3)*
**Attendance while at ULMedX** *(%)*	*45.8 (23.9)*	*64.1 (15.6)*	*27.4 (14.9)*
**Attendance Range** *(%)*	*40–60*	*60–80*	*20–40*
**Duration at ULMedX** *(months)*	*8.1 (5.9)*	*13.8 (1.5)*	*2.4 (1.9)*

Abbreviations, *SD* Standard deviation, % percentage of group which expressed this characteristic, *n* number of participants, *km* kilometres, *PWDO* people who have dropped out

The researchers identified four themes, each with inter-connected sub-themes based on study objectives, data analysis and the TPB (Table 4). ‘Areas for future development’ were also identified.

**Table 4:** Themes and areas for future development

Themes	Sub-Themes	Codes
**1. Support at ULMedX**	***Professional***	GP recommended, professional trainers, very interactive, easy to understand, reassurance from ULMedX doctor, non-judgemental.
	***Family***	Encouragement, accountability
	***Peer***	Sociable, encouraging peers, friendly atmosphere, meeting people with similar disease, seeing other participants improve.
**2. Benefits from ULMedX**	***Physical Health***	Feeling fit, ease at daily movements, health improvements
	***Mental Health***	Feeling good, increased energy, increased alertness, overcome fear of disease, motivation for fitness, reduced anxiety.
**3. Motivation to attend ULMedX**	***Attitude***	Beneficial to health
	***Behaviour***	Coping ability, stress relief, being monitored, exercise in group, confidence, positive environment, excellent facilities, started exercise at home, encouraged
**4. Barriers towards ULMedX**	***Self-awareness***	Inability to do exercises
	***Physical barriers***	Post exercise pain, unsuitable exercises, travel expense, distance to travel, lack of transport, laziness, parking, commitment to other work, time of the classes, injury
**Areas for future development**	Evening classes, psychological support, advance classes, more variety, more educational seminars, new members start as their own group.	

### Support at ULMedX

#### Professional

Being supervised by staff improved both long-term attenders and PWDO experiences at ULMedX. However, the nuance and diversity amongst accounts are apparent as many participants defined ‘adequate monitoring’ differently. The reports suggest that the definition may be influenced by the individual’s previous experience in EBCR programmes, the perceived staff expertise, and the individual’s current concerns about exercising in community-based settings.

*“{Previously in hospital-based EBCR programme} we were wired to the computer and they could see each person, what their pulse rate was and if your pulse was too high, they’d come around and tell you to slow down and so it was very much a 1-1… whereas here, there’s a very big crowd…, it’s kind of left to yourself to monitor yourself and no one takes your pulse at any time here. So, I see that as a negative.” (PWDO-002).*

Another participant (LT-attender) considered the current level of supervision appropriate, stating;

*“I did feel paranoid but then, I actually looked and saw they had a defibrillator and I kind of said, ‘Okay, I’m safe. If I have a heart attack or I have an issue, these people know what they’re doing and I’m in good hands and that had a huge impact on me in coming back and staying.” (LT-001).*

Guidance and support from staff encouraged participants to adhere to the programme. Many stated that they were more likely to initiate exercise or continue with the programme as a result;

*“…I’m not disciplined enough to do this on my own. I needed somebody to encourage me.” (LT-001).*

#### Family

Thirteen out of the fourteen participants stated that their families were supportive of them attending the EBCR programme and that;

*“It was wise to go to it.” (PWDO-004).*

Participants’ family members would enquire when they skipped a session. Partners, siblings, and children would say things like;

*“Oh, why aren’t you going? When are you going back?” (PWDO-003).*

Some family members would take over family caregiving responsibilities to help the participants attend the programme, with one individual stating;

*“The mornings that I say, ‘aw I don’t know if I’ll do the ULMedX programme’, he’ll say {their partner} ‘no I’m on call for Sarah, go to your programme’.” (LT-001).*

Only one participant (PWDO) stated;

*“They left it up to me really.” (PWDO-002).*

Many participants also found it very encouraging when their family members told them they noticed positive changes in their mood and behaviour;

*“They told me the more physical exercise I do, the more better humour I’m in.” (LT-001).*

Some would make comments like;

“*oh god mam you’re looking so well and you’re feeling so well, you’re in better form.” (LT-003).*

#### Peer

The opportunity to talk after class and engage in participant-led discussions helped many individuals feel less apprehensive about exercising and spurred them to continue engaging in the programme;

*“You can talk to your GP or your wife but there’s nothing like talking to a man whose gone through it all and felt the little things you felt.” (PWDO-005).*

Being surrounded by students inside the university setting was also recognised as a motive for adherence by both long-term attenders and PWDO;

*“You see them all out there and there’s kids and the rugby team and there’s people with disabilities who all come in and it’s like, everyone is here exercising. You just feel really connected to life. I wouldn’t like it in just a village hall in Feakle. I wouldn’t enjoy it as much.” (LT-004).*

Both long-term attenders and PWDO reported feelings of comfort and reassurance when speaking with other individuals who also had first-hand experience of the physical and mental perturbations whilst going through cardiac rehabilitation;

*“It’s almost like an AA meeting. You get to speak to people about issues that yes, you can go and speak to your doctor about, but your doctor is going from what he’s read in the book, he’s not going by first-hand experience.” (LT-002).*

Listening to other participants detail what to expect at different stages of the rehabilitation journey was a prominent feature of this support many participants valued. Participants noted they then felt less worried and more willing to try things;

*“I have learned an awful lot from the other participants about what to expect as normal after heart surgery. I didn’t know what to expect in recovery. So, when I met other participants who’ve had similar type issues, I’ve kind of learned, oh yeah, I still have atrial fibrillation but another lady who had similar surgery to me she has it too. So, don’t worry about me.” (LT-001).*

Seeing other ULMedX participants of a similar age and ability apply themselves and progress over time was very encouraging for many participants. These observations directed awareness to their own exertion levels and offered an image of ‘what could be’ if willing to work hard, with two individuals stating;

*“…I can see people going, getting faster. It’s very encouraging.” (LT-003).*

*“… I’m motivated watching other people working.” (LT-002).*

The motivational aspects of group training identified here do not reflect every experience at ULMedX and may be considered maladaptive for those with lower physical capabilities struggling to keep up, with a PWDO stating;

*“Even though they say, ‘do it at your own pace’, you can’t really because you’re in a group.” (PWDO-002).*

### Benefits from ULMedX

#### Physical health

The adverse effects of cardiac treatment interfered with some individuals’ ability to look after their families and engage in recreational activities. The EBCR programme helped many participants regain their physical functioning and return to normal life;

*“When I started ULMedX, I couldn’t carry shopping, I couldn’t push a large shopping trolley…Then gradually from 3 to 4 months, I progressed from not being able to carry shopping bags, to being able to carry the shopping bags. To not being so breathless. To not being so afraid.” (LT-001).*

#### Mental health

Some participants detailed having difficulties with their mental health prior to attending ULMedX. Cardiac treatment was reported to provoke chronic symptoms of anxiety or depression and low self-confidence for many, with one individual stating;

*“It’s not really until 12 months [post treatment], that’s more when the depression starts to kick in, because you’re kind of going along and saying ‘is this it? Is this [what] life has to offer for me?’. “(LT-002).*

For some, attending ULMedX simply made them ‘feel good’ (PWDO-003). Others noted it helped them come out of seclusion;

*“This really got me out of my shell.” (LT-007).*

Some PWDO decided to participate in sport and or train at their local gyms instead of attending the ULMedX programme. The initial training and support at ULMedX helped them reach a point where they felt confident exercising independently;

“*When you find yourself back on your feet and you’re perfect, there might be a tendency to say okay now, I don’t have to keep going to this medical thing.” (PWDO-005).*

*“I just changed my addiction [into] a positive spin and exercised, where I attend the gym here daily and I suppose, I would have not gotten into this if it wasn’t for that programme.” (PWDO-004).*

### Motivation to attend ULMedX

#### Attitude

Many participants already had a positive attitude towards exercise and understood the importance of lifestyle management before attending ULMedX. They sought help from ULMedX as they found it too challenging to do on their own;

“*With a young family, I didn’t want to die”. (PWDO-006).*

Other long-term attenders also gave similar report. The emotional and physical ‘cost’ of the aftermath was enough to motivate many to engage in the programme;

*“I couldn’t afford to be the way I was, I needed to get my life back.” (LT-001).*

The financial cost of being unwell was also made apparent;

*“I’ve lost so much money anyway from being sick. I was working before, I’ve lost a lot of students and you’re not able to work and yeah, it’s hard.” (LT-004).*

#### Behaviour

For some, ULMedX was the first opportunity to initiate exercise and build better behaviours in a safe, supportive environment;

“*It makes you start, that’s the single most important part of it. It makes you believe that you can do stuff that otherwise you were either afraid to do or you thought you shouldn’t have been doing.” (LT-002).*

### Barriers towards ULMedX

#### Self-awareness

It was common for some participants to ruminate on the exercises they could not yet execute and, as well asthe post-exercise pain. Some felt that the programme was not suitable for them, and this resulted in one individual dropping out;

*“I think it was just a bit too much for me.” (PWDO-004).*

#### Physical barriers

Friends of LT-attenders and PWDO who were also eligible to attend ULMedX felt that the morning classes were too difficult to attend due to traffic and other work commitments;

*“Tommy would be interested in coming, but he can’t get the time off work.” (LT-002).*

Greater travel distances and associated expenses negatively influenced the ability to attend ULMedX. PWDO travelled 19.09 km more on average than the long-term attenders;

*“It was the distance and expenses with the getting there. The cost of getting there. Other than that, I would be still continuing to attend.” (PWDO-004).*

This may have been a major barrier for PWDO to attend ULMedX however, no statistical analysis was conducted to see if there was a significant difference between groups.

Family responsibilities also influenced adherence, with one PWDO stating this was the ultimate reason as to why they dropped out;

*“My thing was my husband was very sick and I had to look after him.” (PWDO-003).*

Some felt that ULMedX might not be suitable for them due to their age;

“*I think it’s a bit much for perhaps older people or those that maybe haven’t got the physical strength.” (PWDO-004).*

Chronic injuries or illnesses were also considered major barriers to adherence. Four out of the seven PWDO had injuries or CVD-related problems that required attention and could no longer attend ULMedX.

### Areas for future developments

#### Programme progression

Modifications to the programme progression to caters to all abilities was a common suggestion. For some, the first few sessions were fatiguing and debilitating, with one individual stating;

*“I was exhausted. I couldn’t drive home. I had to go sleep in my car.” (LT-003).*

Participants suggested including talks from long-term attenders at induction. LT-attenders could briefly talk about their own recovery and discuss what beginners should expect from ULMedX. Delaying the inclusion of new participants into the bigger group (with long-term attenders) was also suggested;

*“I think in some ways… the new group would even benefit from coming in for a week or two of just them and then integrate them into the big group.” (LT-002).*

#### Psychological support

Participants suggested that the programme should offer more information about mental health. Educating all the participants on the benefits of exercise for positive mental health was also suggested;

*“I do think that there needs to be more stuff on the mental side of it because as I said, I think people-, they just don’t know. Nobody tells them.” (LT-002).*

#### Class timetable

PWDO and long-term attenders recommended tailoring classes to suit different abilities. They suggested this modification will allow individuals to work at their desired intensity and would offer more scope for progression;

*“I was progressing a little bit faster… so mainly, I did feel a little bit held back.” (PWDO-004).*

The addition of evening classes was another common recommendation to combat common barriers to adherence;

*“An evening class where somebody that is working could come and do the class. I think it would be huge.” (LT-001).*

## Discussion

The current study offers an exploration into the experiences of both long-term attenders and PWDO of a community-based EBCR programme in Ireland. Three inter-related attributes shaping participant experience and commitment to attendance were established. These were (a) social support surrounding engagement (b) perceived outcomes resulting from participation and (c) the practical issues with attendance. Results were in line with TPB propositions which were that individuals were more likely to have had a better experience and commit to the programme if they believed involvement would benefit their physical and mental health, increase their exercise motivation by engendering a positive attitude to exercise, and that the ability to attend was within their control [22, 23].

The social and medical support associated with ULMedX had a major impact on the participants’ experience and commitment to the programme. This support generated feelings of exercise competence and safety. A major barrier to exercise participation, particularly in older adults, is fear that exercise will exacerbate existing conditions [24]. Although some preferred the idea of ability-based classes, mixed ability and cooperative groups encourages a mastery-based climate and are core theoretical strategies shown to improve self-efficacy as proposed by Bandura [25]. This is particularly important as barrier self-efficacy [26] has been recognised as crucial for long-term PA adherence in EBCR programmes [27]. Support from fellow ULMedX members encouraged commitment, favouring previous work on exercise motives in EBCR [28, 29]. High levels of family support also assisted with commitment, in line with previous research [30, 31].

Perceived mental health improvements were reported to result from participation at ULMedX, supporting other works examining EBCR programmes [29, 32]. The participants’ strong intention to engage in the programme to ‘get their life back’ post-cardiac treatment highlights the drastic change in the quality of life many people experience after cardiac treatment [29, 32]. In addition, the ‘feel-good factor’ associated with engagement is consistent with evidence that exercise training can significantly alleviate symptoms of anxiety among individuals with CVD [33].

Greater travel distance and associated costs were major barriers to commitment for long-term attenders and the primary barriers to engagement in PWDO. Studies have shown that as the length of travel rises over 30 min, individuals are less likely to engage in EBCR programmes [34, 35]. Family responsibilities acted as a barrier for some, and the evidence confirms family factors' significant effects on  on exercise adherence [23]. Additional barriers including injury, working status and the lack of parking facilities are similar to findings in other studies [36, 37]. Nevertheless, participants affected by practical barriers still held ULMedX in high regard, supporting the TPB proposition that perceived behavioural control can directly influence behaviour despite intention and attitude [22].

## Conclusion

The findings indicate that EBCR programmes be reviewed so that their structure centres on the participants’ motivations to engage in the programme. The results of this study support previous findings surrounding factors interfering with adherence and common motives in those participating in long-term cardiac rehabilitation. The findings also indicate that this ECBR programme in Ireland requires the introduction of new strategies to resolve practical barriers to adherence, such as home-based programmes using digital resources [38–40].

### Strengths and limitations

The ‘semi-structured’ nature of the interviews and the questions chosen enabled us to capture a variety of experiences at ULMedX to date [41]. In addition, this research was also able to outline the attitude participants have towards ULMedX and EBCRs in general, including PWDO.

In turn, some limitations of the study should be considered. The interaction between the primary researcher, who consistently engaged in programme delivery and interacted with members, and participants (mainly long-term attenders), who strongly desired to attend the only local EBCR programme, may have produced an understanding which depicted the programme in an overly positive light. To minimise this social desirability bias, the interview scripts included both open and closed questions and specifically indicated that both positive and negative feedback was welcomed. Factors like EBCR programme location, resources, and public transport strongly influence the relevance of our findings however, the results may still be applicable to other programmes in Ireland being set up in third level institutions or community settings.

### Areas for future development at ULMedX

Several issues must be addressed to  improve the overall adherence and most importantly, prevent early drop-out at ULMedX. In an effort to achieve this, the following suggestions have been generated in light of this study’s results:

- Talks at induction from long-term attenders. It was suggested that this strategy might reduce initial levels of intimidation. However, this is a novel idea that would require additional planning and organisation and this suggestion has been made by participants in similar programmes, supporting its plausibility [30, 42].
- Initially separating beginners and long-term attenders. It was suggested that this strategy would reduce initial levels of intimidation and that it would give ensure a greater understanding of the exercise education element.
- Regular check-ins with individuals who have suffered from an injury. As injury ultimately resulted in multiple individuals dropping out of the programme, strategies like this may help re-engage participants when fully recovered.
- Providing information about the impact chronic illnesses can have on mental and physical health. Information will be provided through newsletters and educational seminars.
- Individualised training to help participants deal with caregiver stress.
- Offer class timetables to support working life and opportunities to attend ability-based sessions. For those with low physical fitness levels, the provision of an alternative exercise class may encourage their long-term commitment to attend.

### Research

It is plausible that unique settings like ULMedX within the community, as opposed to the hospital, and directly accessible to general practitioners could be seen to promote a sense of normality and give CVD patients confidence to continue to exercise post-hospital-based EBCR completion. Further research comparing the effects of different EBCR settings on participant experience is indicated in this area. In addition, quantitative outcome measures are needed to determine significant psychological and physical health changes at ULMedX [12].

## Data Availability

The datasets used and/or analysed during the current study are available from the corresponding author on reasonable request.
